# The Differential and Interactive Effects of Aging and Mental Fatigue on Alpha Oscillations: A Resting-State Electroencephalography Study

**DOI:** 10.3390/brainsci15060546

**Published:** 2025-05-22

**Authors:** Xiaodong Yang, Kaixin Liu, Lei Liu, Yanan Du, Hao Yu, Yongjie Yao, Yu Sun, Chuantao Li

**Affiliations:** 1 School of Psychological and Cognitive Sciences, Peking University, Beijing 100871, China; beckyxd1991@pku.edu.cn (X.Y.);; 2 Naval Medical Center, Naval Medical University, Shanghai, 200433, China; 3 School of Psychology, Qufu Normal University, Qufu 273165, China; 4 Department of Biomedical Engineering, Zhejiang University, Hangzhou 310027, China

**Keywords:** alpha oscillation, resting-state EEG, aging, mental fatigue, functional connectivity

## Abstract

**Background:** Both aging and cognitive fatigue are significant factors influencing alpha activity in the brain. However, the interactive effects of age and mental fatigue on the alpha spectrum and functional connectivity have not been fully elucidated. **Methods:** Using resting-state EEG data from an open-access dataset (younger: *N* = 198; older: *N* = 227) collected before and after a 2 h cognitive task block, we systematically examined the effects of aging and mental fatigue on alpha (8–13 Hz) oscillations via an aperiodic-corrected power spectrum, the weighted phase lag index (wPLI), and graph theory analysis. **Results:** In both spectral power and network efficiency, mental fatigue primarily modulates low alpha in younger individuals, while high alpha reflects stable age-related changes. The aperiodic offset and exponent decrease with age, while mental fatigue leads to an increase in the exponent. Notable interactions between age and mental fatigue are observed in low-alpha power, the aperiodic exponent, and the network efficiency of both low- and high-alpha bands. **Conclusions:** This study provides valuable insights into the differential modulation patterns of alpha activity by age and mental fatigue, as well as their interactions. These findings advance our understanding of how aging and mental fatigue differentially and interactively shape neural dynamics.

## 1. Introduction

Mental fatigue, a psychophysiological state of deterioration following sustained or high-intensity cognitive demands, is characterized by attentional lapses, diminished decision-making capacity, and elevated task error rates [1,2,3]. Older adults appear more likely to report cognitive decline symptoms resembling mental fatigue, such as working memory deterioration [4] and attentional deficits [5]. Studies have demonstrated that cognitive fatigue impacts behavioral performance in older adults, manifesting as prolonged reaction times in complex tasks compared to in younger individuals [5,6]. However, the relationship between cognitive fatigue and aging, and specifically how these factors interact in modulating neural oscillations, has not been systematically investigated.

Electroencephalography (EEG) has emerged as an effective technique for detecting cognitive fatigue and elucidating its neural mechanisms. Alpha rhythm (8–13 Hz), the most prominent oscillatory activity during eyes-closed rest, is critically implicated in attentional gating and cognitive control [7]. Alpha activity is modulated by aging and various physiological–psychological states [8]. With advancing age, resting-state alpha power and functional connectivity are typically diminished [9,10,11]. Mental fatigue also induces significant alpha modulation: prolonged cognitive tasks lead to cerebral fatigue and reduced vigilance [3,12,13,14]. Existing studies have found a link between resting-state alpha and subsequent cognitive performance [15,16]. Auer et al. (2024) demonstrated that alpha-power modulations correlate with memory task performance in healthy older adults [13]. Furthermore, substantial evidence indicates that hypoxia [17], sleep deprivation [18], and pathological aging [9] typically lead to decreased alpha activity and functional connectivity. The convergent evidence positions alpha rhythm as a promising candidate neuromarker for detecting cognitive impairment.

Recent studies have subdivided EEG frequency bands into narrower ranges, with the alpha band further partitioned into low alpha (8–10 Hz) and high alpha (10–13 Hz) based on their unique characteristics [9,14,19]. Li et al. (2020) found that the alpha1 sub-band demonstrates superior sensitivity to fatigue detection [14]. Sun et al. (2014) applied alpha1-specific features for mental fatigue classification, achieving high prediction accuracy [20]. Notably, emerging evidence suggests that alpha-power changes in the spectral domain may reflect a composite of periodic oscillatory components and aperiodic neural noise [21,22]. The aperiodic component dynamically shifts with aging, task demands, and cognitive states, potentially confounding alpha-power measurements [23,24]. Therefore, whether aging and cognitive fatigue exert distinct or overlapping effects on periodic and aperiodic neural activities remains to be elucidated.

Aging profoundly impacts not only local alpha power but also the functional connectivity of global brain networks. Older adults typically exhibit reduced functional connectivity during cognitive tasks, particularly between higher-order cognitive regions such as the prefrontal and parietal cortices [25]. Recent studies leveraging graph theory, a robust analytical framework for network neuroscience, have revealed age-related declines in brain network organization and efficiency. Specifically, older adults demonstrate diminished global and local network efficiency during cognitive tasks, characterized by compromised interregional collaboration and inefficient information processing [26].

In older adults, compensatory task strategies often interact with inherent cognitive decline, leading to ambiguous interpretations of behavioral and electrophysiological outcomes [27]. Previous studies have predominantly focused on task-related brain activity alterations [26,27,28,29,30]. However, the magnitude of task-induced alpha-power and network efficiency modulations may be confounded by the task type, difficulty, and individual variability (e.g., age, vigilance). Resting-state EEG (rs-EEG) provides an objective metric for quantifying spontaneous neural activity, circumventing confounds from task demands or strategic biases. Critically, recent evidence demonstrates that rs-EEG metrics exhibit sustained post-task alterations following cognitive exertion (task aftereffect), positioning rs-EEG as a powerful tool to dissociate aging-related long-term neural adaptations from fatigue-driven short-term neural perturbations [31,32].

While numerous studies have separately examined the effects of aging and cognitive fatigue on alpha activity, a critical gap remains: aging and cognitive fatigue may differentially modulate spontaneous neural activity. Furthermore, how cognitive fatigue distinctly impacts neural dynamics in younger and older adults remains poorly understood. This study addresses these questions by analyzing a large-scale resting-state EEG dataset through parameterized spectral decomposition using the Fitting Oscillation and One-Over-Frequency Component (FOOOF) algorithm and graph theoretical analysis of functional connectivity based on the weighted phase lag index (wPLI). By characterizing the effects of aging and mental fatigue on alpha activity, we aim to unravel the complex interplay between aging and cognitive fatigue in shaping neural oscillatory mechanisms. We hypothesize that the EEG spectral and connectivity metrics may exhibit age-dependent divergent patterns of change before and after cognitive tasks. By introducing a refined analytical pipeline estimating aperiodic decomposed power spectral and wPLI-based functional connectivity efficiency, we demonstrate its potential to optimize age- or fatigue-related biomarkers and enhanced the understanding of their underlying neurophysiological mechanisms.

## 2. Materials and Methods

### 2.1. Data Sourcing and Description

We utilized a publicly available rs-EEG dataset OpenNeuro dataset: ds005385 (https://openneuro.org/datasets/ds005385, accessed on 14 December 2024) from a prospective cohort investigation into determinants of healthy cognitive aging, which is a part of the Dortmund Vital Study(for further detailed information pertaining to the dataset, please refer to [33]). The dataset consists of resting-state EEG data from 608 adults aged 20–70 years, acquired before and after a 2 h cognitive task battery. Pre- and post-task resting-state acquisitions included 3 min eyes-closed (EC) and eyes-open (EO) conditions, with EC preceding EO to establish arousal and activation baselines, respectively. In order to control the level of wakefulness, the cognitive testing was conducted during the morning at the same time for all participants. The recording followed the same procedure for all participants, and the EC condition was always prior to the EO condition. All procedures adhered to the Declaration of Helsinki, with written informed consent obtained under ethical approval from the Leibniz Research Centre for Working Environment and Human Factors (A93-1).

**Figure 1:**
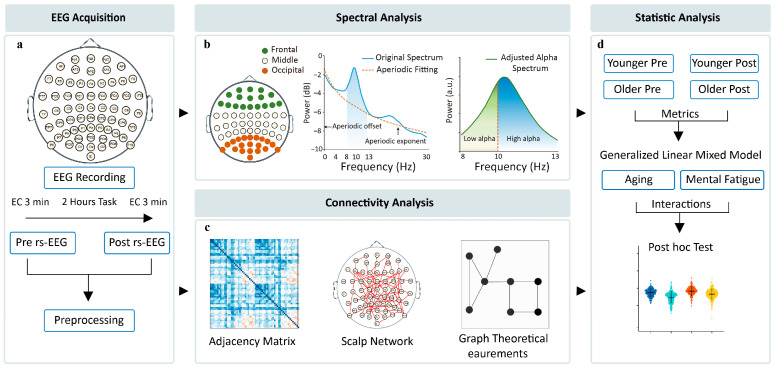
Schematic diagram illustrating the pipeline for (**a**) EEG data acquisition (obtained from the publicly available dataset), (**b**) spectral analysis, (**c**) connectivity analysis, and (**d**) statistical analysis.

EEG data collection utilized a 64-channel ActiCap system (10–20 montage) referenced to FCz, recorded at 1000 Hz via BrainVision amplifiers (BrainProducts GmbH) with online low-pass filtering (250 Hz) and electrode impedances <10 kΩ. The data acquisition and analysis pipeline are illustrated in Figure 1a. A relative technical validation was provided along with the dataset to estimate the EEG data quality [34]. The intra-class correlation (ICC) in the alpha power of 370 participants from this dataset ranged between 0.92 and 0.94 in the EC condition, showing good alpha-power reliability. In another analysis, good short-term and long-term re-test reliability of the EEG microstate was observed in a subgroup of 583 participants from this dataset [35].

### 2.2. EEG Preprocessing

The age classification followed the criteria for cognitive aging research [31]. A total of 11 participants were excluded due to excessive artifacts during preprocessing or insufficient data length after ICA correction, resulting in a final sample of 198 younger adults (20–34 years, M = 27.12 ± 3.92, 66.67% female) and 227 older adults (51–70 years, M = 59.52 ± 5.75, 58.15% female).

A standardized EEG preprocessing protocol was performed using the EEGLab toolbox [36] in MATLAB R2024a (MathWorks, Natick, MA, USA). The data were (1) resampled to 250 Hz, (2) subjected to band-pass filtering (0.5–45 Hz) and notch filtering at 50 Hz to eliminate power-frequency interference, and (3) visually inspected with the artifacts removed manually. Then, (4) the Independent Component Analysis (ICA) was employed to further identify and remove artifacts such as eye movements, blinks, and muscle artifacts [37]; (5) the data were re-referenced to the average of the whole brain EEG signal; and (6) signal pieces were discarded with amplitudes exceeding ± 100 μV and (7) segmented based on the start and end markers to facilitate further analysis.

### 2.3. Spectral Analysis

Spectral analysis was conducted using the FOOOF algorithm toolbox [38] on the log-power spectra in the 1–30 Hz frequency range. The power spectrum for each channel was first computed using the Welch method (6 s Hamming window, 50% overlap). Subsequently, the FOOOF algorithm was applied to decompose the EEG power spectrum into periodic and aperiodic components. The original power spectrum (P) is modeled as the sum of the aperiodic component and periodic components as follows:

(1) $$P=L+\sum_{n=0}^{N}G_{n}$$

where L is the aperiodic component as a 1/f-like function of frequency $\left(F\right)$, and *N* is the number of detected Gaussian peaks $G_{n}$. The periodic component is modeled using Gaussian functions. The formula for the Gaussian peak at each $G{\left(F\right)}_{n}$ is as follows:

(2) $$G{\left(F\right)}_{n}=a*\exp\left(\frac{-{\left(F-c\right)}^{2}}{2*\omega^{2}}\right)$$

where a is the amplitude of the peak, c the center frequency, and ω the bandwidth of the peak. The aperiodic component L can be described by the following formula:

(3) $$L=b-log(K+F^{x})$$

where b is the aperiodic offset, representing the uniform shift of power across frequencies; x is the exponent, reflecting the rate of power decay with increasing frequency; K is the knee parameter; and *F* is the vector of input frequency. The specific settings of the algorithm included peak width limits 1–8 Hz, a maximum of 6 peaks, a peak threshold of 2, and a minimum peak height of 0.1, with “fixed mode” on (i.e., without considering the knee parameter).

### 2.4. Functional Connectivity

To estimate functional connectivity based on electrode channels, the wPLI was used. The wPLI quantifies the phase synchrony between two EEG signals by measuring phase angle differences, effectively minimizing the influence of non-physiological volume-conducted signals [39]. The wPLI calculation process is as follows: First, the selected channels are band-pass-filtered, followed by Hilbert transform to obtain the instantaneous phase (as shown in Equation (3)).

(4) $$S_{x}\left(t\right)=s_{x}\left(t\right)+{i\;\bar{s}}_{x}\;\left(t\right)$$

where $S_{x}\left(t\right)$, represents the analytical signal of the x channel, $s_{x}\left(t\right)$ is the real-time series of the x channel, and ${imag\;\bar{s}}_{x}\;\left(t\right)$ is the Hilbert transform for $s_{x}\left(t\right)$. Then, the complex cross-spectrum between the selected channels, i.e., x and y, is presented by Z(t):

(5) $$Z(t)={S_{x}\left(t\right)S_{y}\left(t\right)}^{*}$$

where ${S_{y}\left(t\right)}^{*}\;$ represents the complex conjugate of $S_{y}\left(t\right)$. Finally, the wPLI is estimated via the imaginary component of the cross-power spectrum:

(6) $${wPLI}_{xy}=\frac{\left|\langle \left|imag(Z)\right|sign(imag(Z))\rangle \right|}{\left|\langle imag(Z)\rangle \right|}$$

where || is the absolute value, 〈〉 represents the mean value, and sign denotes the signum function. The wPLI values range from 0 to 1, and the grand-averaged wPLI across segments is calculated and used for further analysis.

### 2.5. Graph Theory Analysis

The network efficiency of low- and high-alpha sub-bands was calculated using The Brain Connectivity Toolbox [40]. We employed both global and local weighted efficiency metrics to evaluate the transmission efficiency within the network. Global efficiency (Eglob) quantifies a network’s integration capacity, whereas local efficiency (Eloc) assesses its segregation or specialization degree [41]. The connectivity adjacency matrix was calculated using a dynamic thresholding strategy, iterating across a threshold range of 0.2 to 0.45 (step size 0.01) [42]. For each threshold, a weighted matrix was calculated. The global efficiency was calculated as follows:

(7) $$Eglob=\frac{1}{N\left(N-1\right)}\sum_{i\neq j}\varepsilon_{ij}$$

where N is the total number of nodes in the network, and $\varepsilon_{ij}$ is the efficiency between nodes i and j, defined as the inverse of the shortest path. The efficiency metrics range between 0 and 1. Local efficiency is the average global efficiency of all node subgraphs and is computed as follows:

(8) $$Eloc=\frac{1}{N}\sum \left(\frac{1}{N_{G_{i}}\left(N_{G_{i}}-1\right)}\sum_{j\neq k\in G_{i}}\varepsilon_{jk}\right)$$

To mitigate threshold selection biases, an iterative thresholding procedure was employed to compute Eglob and Eloc across the entire threshold range, with their respective mean values subsequently serving as final metrics for statistical analyses [43].

### 2.6. Statistical Analysis

Statistical analysis was conducted to assess the differences in the spectral metrics and graph theory connectivity metrics between younger and older groups before and after the task. Through exploratory analysis of alpha-band topographical distribution, we adopted a simplified whole-brain electrode grouping, frontal (anterior), middle (central), and occipital (posterior) for subsequent analyses to examine group-level alpha dynamics. As shown in Figure 1, spectral metrics include high-alpha power, low-alpha power, aperiodic offset, and aperiodic exponent. Generalized Linear Mixed Models (GLMMs) were applied across three brain regions (frontal, middle, and occipital) to evaluate the fixed effects of aging (younger vs. older groups), mental fatigue (pre-task vs. post-task), and their interaction, while incorporating random effects to account for interindividual variability. The models utilized a beta distribution family with a logit link function to accommodate the skewed distribution and continuous nature of the dependent variables. Statistical significance was set at an uncorrected threshold of *Pr* < 0.05. Post hoc subgroup comparisons (e.g., between age groups or task states) were performed using Wilcoxon rank-sum tests with *p* < 0.05 (Bonferroni-corrected) considered statistically significant. GLMM analyses were conducted using the glmmTMB package (v1.1.10) in R (v4.3.0). Non-parametric tests were implemented using the stats package (v4.4.2) in R (v4.3.0).

## 3. Results

### 3.1. Age- and Task-Related Differences in Spectral Metrics

**Figure 2:**
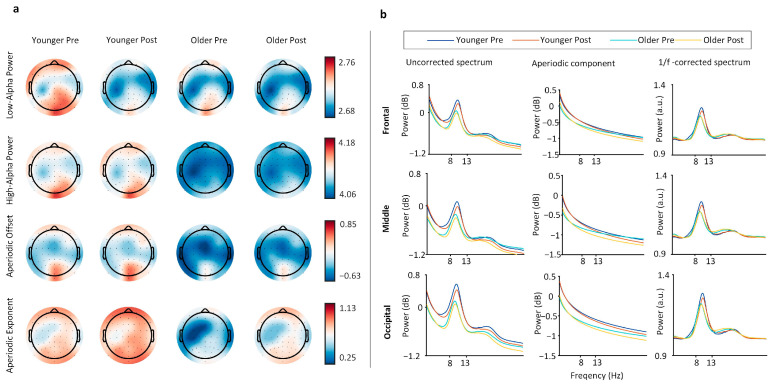
Brain topographies and spectral results of alpha power across different groups and task states. (**a**) Brain topographies for 1/f-corrected low-alpha power, high-alpha power, aperiodic offset, and aperiodic exponent. (**b**) Average spectra for frontal, middle, and occipital regions, showing uncorrected power (left panel), aperiodic components (middle panel), and 1/f-corrected spectra (right panel).

The results of spectral analysis are shown in Figure 2a, which presents the brain topographies for the 1/f-corrected low-alpha power, high-alpha power, aperiodic offset, and aperiodic exponent. Figure 2b shows the average uncorrected power spectra, aperiodic components, and 1/f-corrected spectra for the frontal, middle, and occipital regions.

As shown in Table 1, GLMM analysis of spectral metrics showed significant reductions in low-alpha power under the influence of both age and task factors (all *Pr* values < 0.05), with a significant interaction effect between age and task (all *Pr* values < 0.05). High-alpha power was significantly lower in the older group compared to in the young group (all *Pr* values < 0.05), with no significant task-related changes (*Pr* > 0.05), except for a significant interaction effect in the occipital region (*Pr* = 0.019). Aperiodic offset was significantly lower in the older group (all *Pr* values < 0.05), with significant task-related effects observed only in the frontal region (*Pr* = 0.021). There was no significant interaction effect between age and task for the aperiodic offset (all *Pr* values > 0.05). The aperiodic exponent was significantly lower in the older group (all *Pr* values < 0.05) and significantly increased post-task in both age groups (all *Pr* values < 0.05), with a significant interaction effect between age and task (all *Pr* values < 0.05).

**Figure 3:**
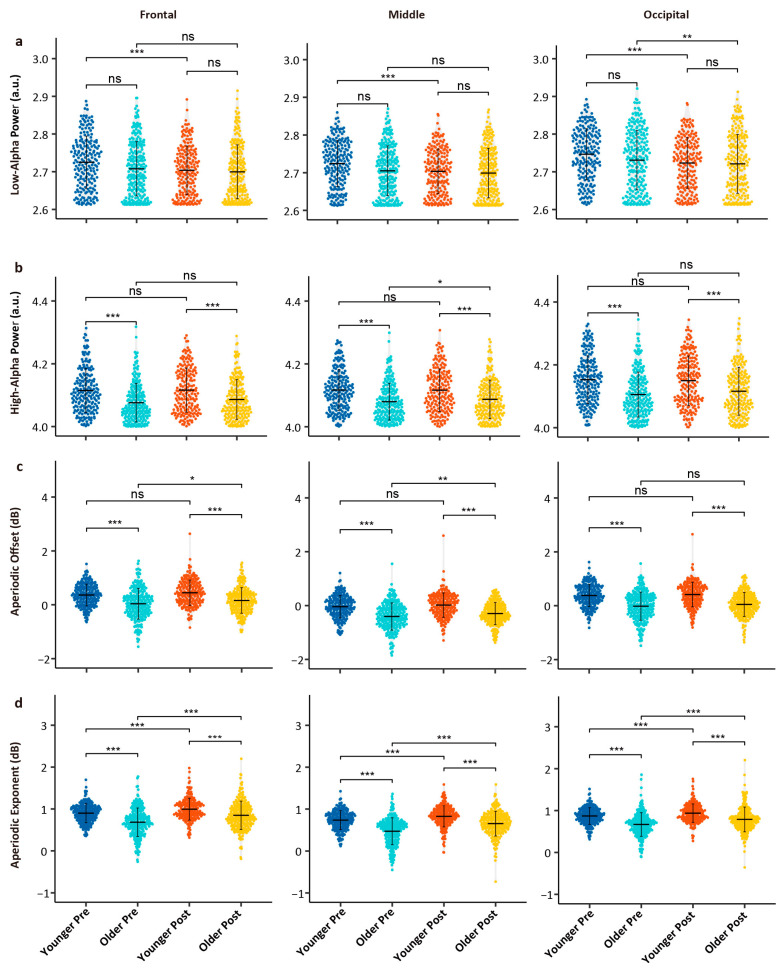
Spectral analysis results. (**a**) Low-alpha power; (**b**) high-alpha power; (**c**) aperiodic offset; (**d**) aperiodic exponent. Note: * indicates there is significance at a significance level of *p* < 0.05; **: *p* < 0.01; ***: *p* < 0.001; ns: no significant difference. This is Bonferroni-corrected.

The Wilcoxon rank-sum test results for spectral metrics are shown in Figure 3. For low-alpha power, no significant differences were observed between the young and older groups before or after the task (all *p* values > 0.05); however, in the young group, low-alpha power significantly decreased post-task across all brain regions (all *p* values < 0.05). In the older group, significant decreases were only observed in the occipital region post-task (*p* = 0.019). For high-alpha power, the older group exhibited lower values than those of the young group before and after the task (all *p* values < 0.05); no significant task-related changes were observed in the young group (all *p* values > 0.05), while the older group showed a significant decrease in high-alpha power in the middle brain region post-task (*p* = 0.018). For aperiodic offset, the older group showed significantly lower values compared to those of the young group before and after the task (all *p* values < 0.05); in the young group, no significant task-related differences were observed, while in the older group, significant decreases were found in the parietal (*p* = 0.017) and middle brain regions (*p* = 0.002). For the aperiodic exponent, the older group showed significantly lower values compared to those of the young group, and both groups showed significant increases in the exponent post-task (all *p* values < 0.05).

### 3.2. Age- and Task-Related Differences in Functional Connectivity

**Figure 4:**
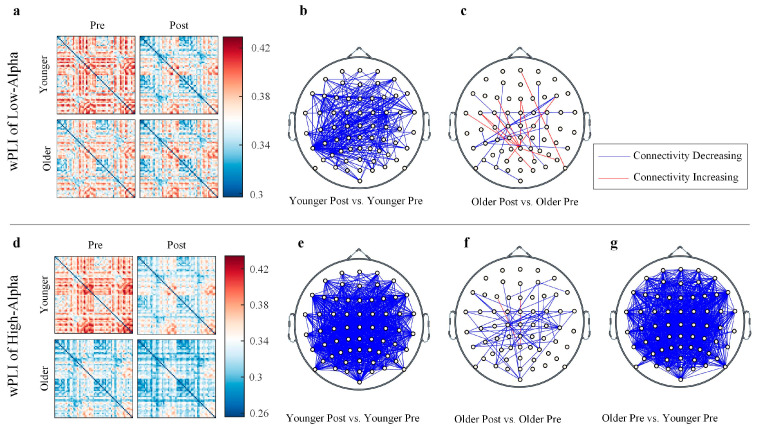
Significant pairwise wPLI connectivity differences between the sub-conditions. (**a**) The low-alpha wPLI connectivity matrix and (**b**–**c**) the significant comparisons. (**d**) The high-alpha wPLI connectivity matrix and (**e**–**g**) the significant comparisons. Non-significant comparisons are omitted. (*p* < 0.05, Bonferroni-corrected). The blue and red edges indicate significant connections between electrodes.

The changes in the functional connectivity in the low- and high-alpha bands, as measured by the wPLI, are shown in Figure 4. For the low-alpha wPLI, the younger individuals showed a significant decrease in connectivity post-task compared to pre-task, whereas older individuals exhibited mixed trends of both increases and decreases in connectivity. For the high-alpha wPLI, the younger individuals showed a significant decrease in connectivity post-task compared to pre-task, while older individuals mainly showed a decrease in connectivity post-task. Pre-task, the older group exhibited significantly lower connectivity compared to the young group, but no significant differences were observed post-task. 

### 3.3. Age- and Task-Related Differences in Graph Theory Metrics

As shown in Table 2, GLMM analysis of the efficiency metrics, Eglob (weighted global efficiency), and Eloc (local efficiency) for the low- and high-alpha bands revealed the significant main effects for task and age, as well as a significant interaction effect (all *Pr* values < 0.05).

**Figure 5:**
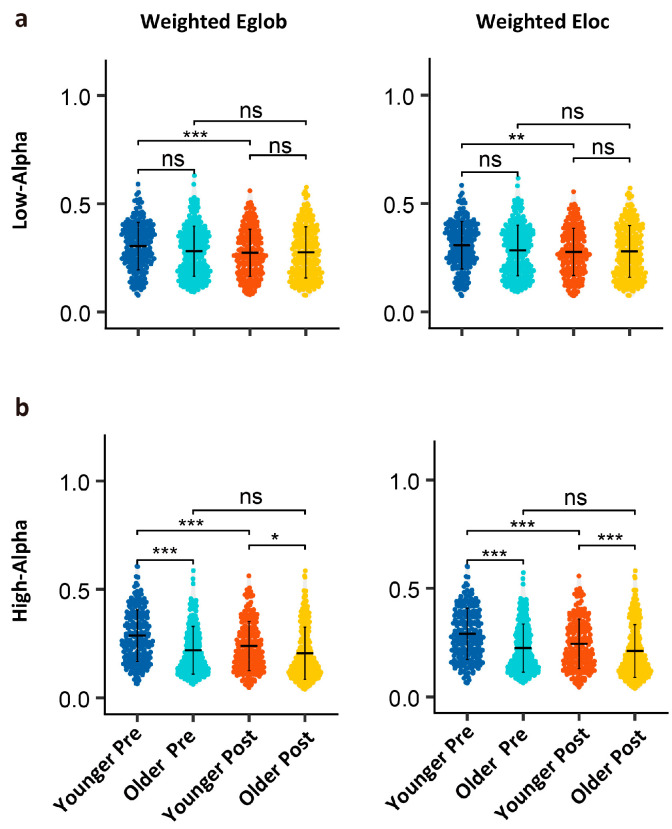
Wilcoxon rank-sum test analysis results for graph theory metrics. (**a**) Network efficiency for low alpha; (**b**) network efficiency for high alpha. Note: * indicates there is significance at a significance level of *p* < 0.05; **: *p* < 0.01; ***: *p* < 0.001; ns: no significant difference. This is Bonferroni-corrected.

As shown in Figure 5, post hoc analysis using wilcoxon rank-sum test revealed no significant differences in the low-alpha network efficiency between younger and older adults across pre-task and post-task conditions (all *p* values > 0.05). A task-induced decline in low-alpha efficiency was observed exclusively in younger adults (all *p* values < 0.01). In contrast, older adults showed no significant pre–post changes in low-alpha power (all *p* values > 0.05). For high alpha, younger adults demonstrated superior baseline network efficiency compared to older adults. In the younger group, significant decreases in high-alpha power were observed post-task (all *p* values < 0.001), whereas in the older group, global weighted efficiency (*p* = 0.005) and local efficiency (*p* = 0.017) showed significant changes.

## 4. Discussion

In this study, based on a large sample of resting-state EEG data, we systematically investigated the effects of age and mental fatigue on alpha power and functional connectivity. The results revealed that mental fatigue primarily modulates low-alpha power and low-alpha network efficiency, with these declines predominantly observed in younger individuals. In contrast, high-alpha power and high-alpha network efficiency, as well as the aperiodic offset, predominantly reflect stable age-related changes. Significant interaction effects between age and mental fatigue were identified for low-alpha power, the aperiodic exponent, and the network efficiency of both low- and high-alpha bands. These findings reveal distinct modulation patterns across different alpha sub-bands and metrics, highlighting the complex interplay between aging and mental fatigue in neural activity.

### 4.1. Power Changes in Alpha Sub-Bands: Aging Drives and Fatigue–Aging Interplay

Our results showed that low-alpha power significantly decreased after cognitive tasks, with a notable interaction effect between age and task effects. Specifically, young individuals exhibited a significant reduction in low-alpha power from before to after tasks, whereas older adults showed a significant decrease only in the occipital region. These findings suggest that changes in low-alpha power are not only cognitively relevant but also modulated by age. In previous studies, the decrease in alpha power induced by tasks is typically associated with cognitive load and cognitive demands [44,45], implying that several hours of cognitive tasks may reduce alpha power through cognitive fatigue and resource depletion.

The difference in low-alpha power before and after tasks is more pronounced in younger individuals, possibly reflecting their better ability to mobilize cognitive resources for dynamic regulation [46]. Moreover, with more marked and flexible suppression of low alpha, the younger showed greater neural plasticity and more efficient neural connections during cognitive tasks [40]. In contrast, older adults experience a decline in their ability to modulate low-alpha power due to reduced neuron numbers and weakened synaptic connections [47], thereby weakening the brain’s response to cognitive tasks [48]. It is plausible that the effect of age on task-related alpha modulation may be a significant process in age-related cognitive decline.

The high-alpha power is significantly influenced by age but not by mental fatigue, aligning with general expectations. High-alpha power is significantly lower in the older group compared to in the younger group, reflecting age-related neurophysiological changes, possibly related to decreased neuronal synchrony in older adults [23]. Previous studies have consistently found that the effect of age is particularly pronounced in high alpha [11,47]. Mental fatigue does not affect high-alpha power significantly, but the interaction between age and cognitive factors in the occipital region suggests differences in the high-alpha responsiveness between older and younger adults. In young adults, increased alpha power prior to cognitive tasks is thought to enhance performance by suppressing task-irrelevant information [49], whereas older adults demonstrate reduced baseline alpha power and delayed post-task recovery [10]. The dynamic plasticity of the brain in older adults is significantly reduced compared to in younger adults, implying that older individuals may not effectively modulate high-alpha power to cope with cognitive load [50].

### 4.2. Decomposing Aperiodic Components in Aging and Cognitive Fatigue

Notably, previous studies remain contentious regarding whether the aperiodic adjusted alpha power consistently reflect age-related changes. After adjusting for aperiodic activity, the peak alpha power decreased in older adults [38], although the age difference in the peak alpha power was reduced after aperiodic adjustment. This seems to differ from the findings of Cesnaite et al. (2023), where no significant relationship between alpha power and age was detected when controlling for the 1/f slope [51]. A reasonable consideration is that these studies did not distinguish between alpha sub-bands, so age-related changes in high alpha may be masked by broader frequency bands [52]. Although aperiodic components can lead to overall shifts in the power spectrum, often overestimating the effect of age, our study found that after FOOOF fitting and correction for aperiodic components, high-alpha power still decreased with age. Our results suggest that high alpha consistently reflects age-related changes.

In addition to periodic rhythmic components, aperiodic components also dynamically change with individual age, task demands, and cognitive states [24,53,54]. Consistent with previous findings [25,38], age-related decreases in the aperiodic offset and exponent were observed. The offset in the older group was significantly lower than in the younger one both pre- and post-task, possibly related to the accumulation of neural noise or the decline in the cortical inhibition–excitation balance [24]. In this study, the offset showed significant differences only in the frontal and middle regions of older adults before and after tasks, but this spatial specificity may be related to experimental conditions, task types, or EEG analysis methods. We found a significant interaction effect between age and task on the aperiodic exponent, where it decreased with age and increased after the task. This may reflect the modulation of different mechanisms: the age-related decrease in the exponent may be due to the weakening of high-frequency activity and reduced cortical excitability [55], while task-related adjustments in the aperiodic component may reflect cognitive resource allocation and fatigue levels [24]. The significant interaction between aging and mental fatigue on the aperiodic exponent suggests that older adults exhibit less task-induced steepening of the aperiodic component compared to younger individuals, despite displaying elevated baseline neural noise. This differential response implies that the age-related cortical excitation–inhibition imbalance may amplify fatigue-induced perturbations in neural noise dynamics.

### 4.3. Functional Connectivity and Network Efficiency

From a global perspective, the decrease in alpha power is also accompanied by a trend of decreased functional connectivity. The wPLI within the low-alpha band is primarily modulated by mental fatigue, while younger and older adults exhibit divergent response patterns. The reductions in the wPLI among younger individuals likely reflect adaptive resource reallocation to meet task-specific attentional demands, aligning with the inhibitory control function of low-alpha oscillations during cognitive engagement [56,57]. In contrast, older adults excited heterogeneous connectivity changes, reflecting age-related differences in coping patterns with cognitive fatigue [58]. These intergroup differences are also consistent with the results of resting-state EEG before and after the task in the present study, which suggests the potential presence of compensatory network reorganization in the elderly population [23,59,60,61,62].

The high-alpha wPLI of older adults is generally lower than that of younger adults across the whole brain, and the task-related decrease is observed only in older adults. This differential trend may be related to the loss of communication efficiency between distant brain regions in the high-alpha frequency band during aging [47,59]. Consistent with power spectral analyses, the decline in low-alpha network efficiency was observed exclusively in younger adults. In contrast, for high-alpha efficiency, aging and mental fatigue demonstrated significant main effects and an interaction effect. These findings align with Bullmore and Sporns’ (2009) framework of economical brain network organization [63], which posits that post-task local network disturbances dynamically reconfigure global and local efficiency. Vecchio et al. (2014) employed weighted matrix graph theory analysis and found that aging affects differences in alpha frequency band connection strength, particularly in the high-alpha band [47]. An fMRI study conducted by Achard and Bullmore (2007) revealed that aging impairs both global and local network efficiency, with the impact on network efficiency being confined to the frontal and temporal cortical regions [64]. Coincidentally, in our earlier results, the aperiodic offset in older adults’ frontal and middle regions is modulated by tasks. An important question for future exploration is whether the increase in aperiodic noise in older individuals is linked to a shared source or mechanism with the task-related decline in network efficiency.

### 4.4. Limitations

This study provides insights into the effects of age and mental fatigue on alpha-power spectrum and brain network dynamics, while there are still constraints that warrant consideration. Firstly, the cross-sectional design precludes the causal inferences or longitudinal tracking of age-related changes. While we controlled for individual variability using methods such as FOOOF, long-term follow-up remains essential. Future studies might leverage the 5-year follow-up cohort of the Dortmund Vital Study dataset, combined with multidimensional data, to elucidate intraindividual neural trajectory evolution and the biological mechanisms underlying fatigue responses. Secondly, the publicly available dataset we analyzed did not include subjective fatigue assessments or cognitive tests results. This limits our ability to establish direct correlations between age-related EEG parameter changes and behavioral manifestations. Given the current dataset constraints, our analysis remains confined to group-level electrophysiological variations, thereby permitting a preliminary investigation into the combined effects of aging and fatigue on neural dynamics. In future experimental paradigms, we plan to incorporate psychological state measurements to further validate the correlation between assessments of psychological states and EEG changes.

## 5. Conclusions

In this study, we systematically explored the effects of age and mental fatigue on the power of low-alpha and high-alpha frequency bands, functional connectivity, network topology, and aperiodic components, using power spectral analysis, functional connectivity, and graph theory. We found that low alpha is mainly modulated by task-induced mental fatigue, whereas high alpha primarily reflects stable, age-related changes. Both mental fatigue and aging contribute to decreased network efficiency in both low- and high-alpha bands. Our findings revealed differential modulation patterns of age and mental fatigue, as well as their interactions, and contribute to a deeper understanding of the neurobiological basis of age-related cognitive decline.

## Figures and Tables

**Table 1:** Statistical analysis results of fixed effects for power spectrum metrics.

			Task			Age		Interaction		
		*z* Value	*Pr* (>|*z*|)		*z* Value	*Pr* (>|*z*|)		*z* Value	*Pr* (>|*z*|)	
Low-alpha power	F	−7.685	<0.001	***	−2.570	0.010	*	3.513	<0.001	***
	M	−8.555	<0.001	***	−3.112	0.002	**	4.425	<0.001	***
	O	−8.551	<0.001	***	−2.170	0.030	*	3.662	<0.001	***
High-alpha power	F	−0.089	0.929		−6.141	<0.001	***	1.367	0.172	
	M	−0.343	0.732		−6.434	<0.001	***	1.894	0.058	#
	O	−1.203	0.229		−6.172	<0.001	***	2.344	0.019	*
Aperiodic offset	F	2.303	0.021	*	−7.123	<0.001	***	1.089	0.276	
	M	1.877	0.061	#	−8.448	<0.001	***	1.575	0.115	
	O	1.083	0.279		−8.895	<0.001	***	1.264	0.206	
Aperiodic exponent	F	4.378	<0.001	***	−7.123	<0.001	***	2.310	0.021	*
	M	4.886	<0.001	***	−8.448	<0.001	***	3.876	<0.001	***
	O	4.028	<0.001	***	−8.895	<0.001	***	2.270	0.023	*

Note: F, M, and O refer to the three brain regions: frontal, middle, and occipital. * indicates there is significance at a significance level of Pr < 0.05; **: Pr < 0.01; ***: Pr < 0.001; #: marginally significant (Pr < 0.1), uncorrected.

**Table 2:** Statistical analysis results of fixed effects for weighted efficiency.

		Task			Age			Interaction		
		*z* Value	*Pr* (>|*z*|)		*z* Value	*Pr* (>|*z*|)		z Value	*Pr* (>|*z*|)	
Low-alpha	Eglob	−4.739	<0.001	***	−2.165	0.030	*	2.784	0.005	**
	Eloc	−4.587	<0.001	***	−2.216	0.027	*	2.845	0.004	**
High-alpha	Eglob	−6.912	<0.001	***	−6.049	<0.001	***	3.158	0.002	**
	Eloc	−6.432	<0.001	***	−5.786	<0.001	***	2.893	0.004	**

Note: * indicates there is significance at a significance level of *Pr* < 0.05. **: *Pr* < 0.01 and ***: *Pr* < 0.001, uncorrected.

## Data Availability

The EEG dataset used in this study (OpenNeuro dataset: ds005385; https://openneuro.org/datasets/ds005385, accessed on 14 December 2024)) was obtained from the Dortmund Vital Study [33]. All procedures adhered to the Declaration of Helsinki, with written informed consent obtained under ethical approval from the Leibniz Research Centre for Working Environment and Human Factors (A93-1).
